# Primary rat LSECs preserve their characteristic phenotype after cryopreservation

**DOI:** 10.1038/s41598-018-32103-z

**Published:** 2018-10-02

**Authors:** Viola Mönkemöller, Hong Mao, Wolfgang Hübner, Gianina Dumitriu, Peter Heimann, Gahl Levy, Thomas Huser, Barbara Kaltschmidt, Christian Kaltschmidt, Cristina I. Øie

**Affiliations:** 1 Biomolecular Photonics, Department of Physics, Bielefeld University, Universitätsstr. 25, 33615 Bielefeld, Germany; 2 Faculty of Health Sciences, Department of Medical Biology, Vascular Biology Research Group, UiT-The Arctic University of Norway, 9037 Tromsø, Norway; 3 Cell Biology and Molecular Neurobiology, Department of Biology, Bielefeld University, Universitätsstr. 25, 33615 Bielefeld, Germany

## Abstract

Liver disease is a leading cause of morbidity and mortality worldwide. Recently, the liver non-parenchymal cells have gained increasing attention for their potential role in the development of liver disease. Liver sinusoidal endothelial cells (LSECs), a specialized type of endothelial cells that have unique morphology and function, play a fundamental role in maintaining liver homeostasis. Current protocols for LSEC isolation and cultivation rely on freshly isolated cells which can only be maintained differentiated in culture for a few days. This creates a limitation in the use of LSECs for research and a need for a consistent and reliable source of these cells. To date, no LSEC cryopreservation protocols have been reported that enable LSECs to retain their functional and morphological characteristics upon thawing and culturing. Here, we report a protocol to cryopreserve rat LSECs that, upon thawing, maintain full LSEC-signature features: fenestrations, scavenger receptor expression and endocytic function on par with freshly isolated cells. We have confirmed these features by a combination of biochemical and functional techniques, and super-resolution microscopy. Our findings offer a means to standardize research using LSECs, opening the prospects for designing pharmacological strategies for various liver diseases, and considering LSECs as a therapeutic target.

## Introduction

The liver is the largest organ in the human body, having essential functions related to maintaining homeostasis and metabolic integrity^1,2^. Liver disease is one of the leading causes of morbidity and mortality worldwide^3–5^. For decades, an immense effort has been undertaken to investigate the mechanisms behind various liver diseases and to develop therapeutic avenues. Despite great strides, many liver disease mechanisms have remained elusive^3,6^. In recent years, the non-parenchymal cells of the liver have gained increasing attention for their potential role in the development of liver disease^7–9^. Among these, the liver sinusoidal endothelial cells (LSECs) are the most abundant non-parenchymal cells, and play a fundamental role in maintaining liver homeostasis^10^. LSECs, the most effective scavengers of blood-borne waste macromolecules in the body^10,11^, form the walls of the hepatic sinusoids and represent a highly specialized type of endothelial cells whose plasma membrane is perforated by numerous nanosized pores, or fenestrations^11–13^. These fenestrations, which range between 50 and 300 nm in diameter under normal physiological conditions, facilitate the bi-directional transfer of substrates between the blood and the underlying hepatocytes^12^. Liver injury coincides with drastic alterations in the LSEC phenotype, resulting in the loss of fenestrations and the formation of a basement membrane^13,14^. LSECs have been reported to play a role in regulating sinusoidal flow^15^, liver regeneration^16,17^, hepatic complications such as hepatitis, fibrosis and cirrhosis^18^, liver immune regulation^14,19,20^, and age-related conditions^21–23^.

Current protocols for isolation and cultivation of LSECs rely on freshly isolating the cells directly from the liver, which must be cultured shortly after isolation due to their rapid dedifferentiation. Their most important *in vivo* features, scavenging function and fenestrations, are severely decreased or disappear completely in LSECs that are kept in culture for more than 1–2 days^24–26^, in particular in LSECs from small vertebrates like rodents^27^. This is accompanied by a downregulation of LSEC signature genes^28^. In addition, the isolation method is time consuming, meaning that to make the *in vitro* experimental conditions reflect the *in vivo* situation the closest (e.g. morphological/functional changes in response to stimuli/drugs), an entire work day may pass before the actual experiment can be carried out. Maintaining functionally intact LSECs in culture for extended periods of time is presently not possible. Approaches to overcome these limitations, such as developing immortalized LSEC lines (reviewed in^18^), have had minimal success^29–31^. These cells lack a proper phenotypic validation, and display very limited LSEC characteristics. The shortage of a consistent source of LSECs has led to the use of alternative cell models, such as non-hepatic ECs^32–34^. However, alternative EC models lack the fundamental characteristics of LSECs with respect to their most important features in the liver, fenestrations and scavenging function.

To date, no protocols have been reported that enable the cryopreservation of LSECs that retain functional and morphological characteristics upon thawing and culturing. Therefore, we sought to develop a protocol that would allow for the cryopreservation of freshly isolated LSECs with intact phenotype upon thawing, similar to their *in vivo* counterparts. We found that freezing down freshly isolated LSECs (fLSECs) at low concentration is a prerequisite for after-thawing recovery of cryopreserved LSECs (cLSECs) with high viability, and full LSEC-signature features: fenestrations, scavenger receptor expression and endocytic function, on par with freshly isolated cells.

## Results and Discussion

Rat LSECs were isolated using the Percoll gradient and selective adherence method^35,36^. This method usually results in 80–120 million LSECs and >95% purity^11^, enough to cover the needs for all the LSEC experiments ongoing in our lab. The leftover cells are sometimes frozen down in pellets for use in e.g. Western blot, RNA/gene expression analyses, or, in most cases, they are simply discarded. In this study we have investigated whether freshly isolated LSECs (fLSECs) would survive cryopreservation, and if, upon thawing, they might maintain their characteristic morphology and function. Two cell freezing media were used, containing 20% or 90% FBS in addition to 10% and 5% DMSO, respectively. Upon thawing and before seeding, the cryopreserved LSECs (cLSECs) were spun down once to remove FBS and DMSO. Although with various cell lines this step is omitted in order to avoid further mechanical stress on the cells, we found it to be necessary to remove the serum since it was previously shown to be toxic to rat LSECs in culture, having a major negative effect on the cells’ viability and endocytic function^27,37^. However, during this centrifugation step, about 25% of the cells are lost. The viability of the recovered cells was tested by Trypan Blue exclusion. We found that the LSECs cryopreserved in 20% FBS had very high viability after thawing as compared to the cells cryopreserved in 90% serum (>90% vs <50% viability, respectively). Moreover, the viability was also drastically affected depending on the number of cells to be frozen down. Increasing the cell count to more than 4 × 10^6^ cells per cryotube resulted in less than 50% viability upon thawing, and the ability of the cells to adhere to the substrate in culture was dramatically reduced. After optimization of the method, dozens of vials from dozens of animals were thawed and used for various projects. The viability of the cells in these vials was similar to the one presented here. The thawed cLSECs were seeded in serum-free RPMI on plastic or glass surfaces coated with fibronectin, to allow adhesion and spreading. While fLSECs normally adhere to the substrate and fully expand their cytoplasm within 2 h from the time they were seeded^35^, the cLSECs required about 1.5 h for optimal adherence, and an additional 1.5 h for spreading of their cytoplasm.

**Figure 1:**
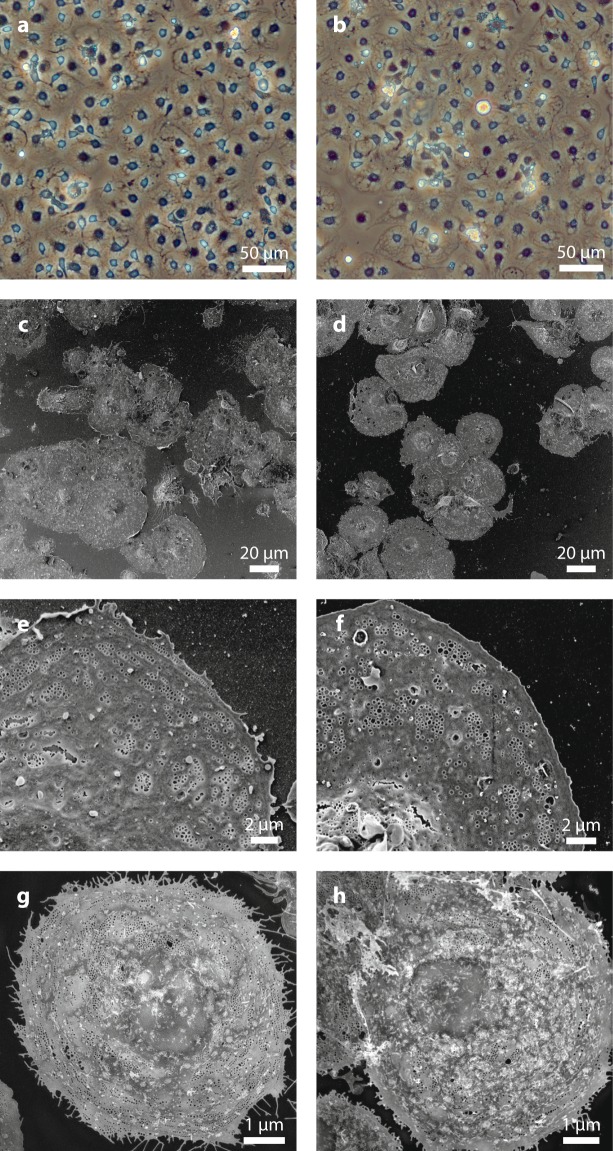
General morphology of fLSECs and cLSECs. In all micrographs, the left panels show images of fLSECs, and the right panels show images of cLSECs. (**a**) and (**b**) light microscopy images displaying the general morphology of the live cell cultures. (**c**) and (**d**) SEM micrographs of large fields of view of the two fixed cultures. The cells had the typical “fried-egg” shape, and an identical size of 29 ± 7 µm for both fLSECs and cLSECs (Statistical details are presented in Table 2 under Materials and Methods). (**e**) and (**f**) High magnification SEM micrographs showing approximately one quarter of an entire LSEC. Numerous fenestrations are visible in both cell conditions, clustered in sieve plates, in smaller groups or standing alone, spread throughout the cytoplasm. (**g**) and (**h**) Maximum intensity Z-projections of 3D-SIM images of an entire LSEC from live cultures of fLSECs and cLSECs, respectively. For visualization of the plasma membrane and fenestrations, the fLSECs were stained with Vybrant DiO, and the cLSECs with CellMask Deep Red.

**Figure 2:**
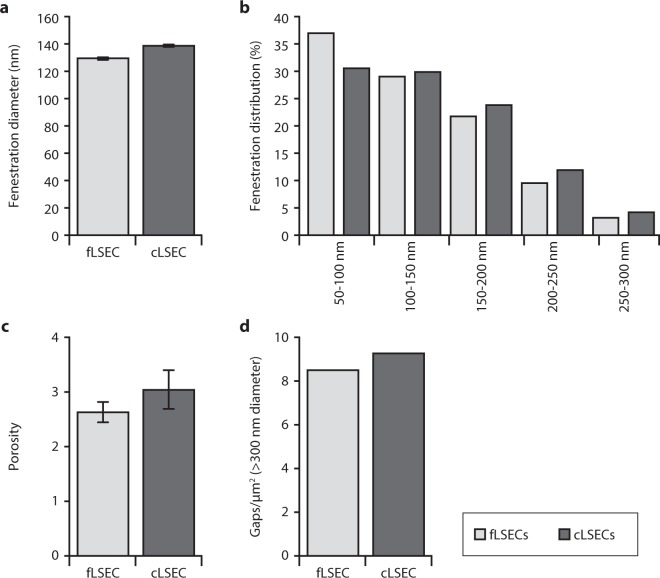
Ultrastructural features of fLSECs and cLSECs. (**a**) Average fenestration diameter. Statistical details are presented in Table 2 under Materials and Methods. (**b**) Frequency distribution of the fenestration diameter in fLSECs (light gray) versus cLSEC (dark gray), respectively. (**c**) Porosity (percentage) = total area covered by fenestrations per total surface area analyzed. (**d**) Number of gaps per µm^2^ (holes larger than 300 nm in diameter). Bars represent mean ± SEM.

The general morphology of both types of cultured cells was assessed by light microscopy (LM) and, for ultrastructural details, by super-resolution structured illumination microscopy (SR-SIM) and scanning electron microscopy (SEM) (Fig. 1). No differences in cell shape or size were observed between the two groups. The cells had the typical “fried-egg” like shape, and an identical diameter of 29 ± 7 µm in both groups (Fig. 1a–d). The unique morphological characteristic of LSECs, their fenestrations, were present in both fLSECs and cLSECs in culture (Fig. 1e–h). They could be observed clustered in sieve plates, spread throughout the cytoplasm in smaller groups, or standing alone. The average diameter of the fenestrae in fLSECs was similar, and not significantly different between the two cell conditions (130 ± 0.2 nm in fLSECs and 139 ± 0.4 nm in cLSECs (reported with standard error of the mean)) (Fig. 2a). Figure 2b compares the distribution of fenestrations in the two cell cultures, grouped in different diameter size ranges. Compared to the fLSECs, the cLSECs had 18% fewer fenestrations with small diameters lying between 50–100 nm. Also, the cLSECs had 27% and 34% more fenestrations with large diameters between 200–250 nm and 250–300 nm, compared to fLSECs. Porosity, defined as the total area covered by fenestrations per total surface area analyzed, was also not significantly altered by cryopreserving the cells (2.63 ± 0.19% versus 3.05 ± 0.35% in fLSECs versus cLSECs, respectively) (Fig. 2c). Fused fenestrations, i.e. adjacent fenestrations which have lost some of the intervening cytoplasm, were not included in the analysis. However, in cLSECs we observed an increase in fenestration distribution in the thicker membranes, close to the nuclear region, as compared to fLSECs (not shown). This nuclear distribution may be due to the fact that the cLSECs required more time for establishment in culture and expansion of their cytoplasm to fully expose the fenestrations in the thin areas of the cytoplasm. Gaps larger than 300 nm were equally observed in both cell groups: 8.5 gaps/µm^2^ in the fLSECs versus 9.3 gaps/µm^2^ in the cLSECs (Fig. 2d).

Preparation of the samples for SEM requires a series of fixation and dehydration steps that can generate artifacts, such as cracks and/or shrinkage of the specimen and alteration of tissue structure^38^. To avoid this, we have also assessed the fenestrations in the two cell groups using super-resolution structured illumination microscopy (SR-SIM). This wide-field nanoscopy technique uses patterned illumination from a coherent light source to convert otherwise unobservable structures below the resolution limit of light microscopy into observable ones by generating difference/beat frequencies called Moiré fringes^39–43^. The reconstructed image then has a resolution two times higher than that obtained by conventional light microscopy, which is well within the average diameter of fenestrations^44^. Compared to SEM, the samples to be imaged by SR-SIM can be wet, meaning that the cells can be observed while in culture medium, and without fixation, thus avoiding dehydration artifacts and providing the greatest potential for live cell imaging^45,46^. Here, we have used 3D-SIM to image fenestrations in live rat LSECs from both freshly isolated and cryopreserved cultures. Similar to the observations from SEM images, LSECs from both cultures expressed numerous fenestrations (Fig. 1g,h). However, due to the limitation of the linear SR-SIM technique, only fenestrations with a diameter of 100 nm or more are fully resolved.

**Figure 3:**
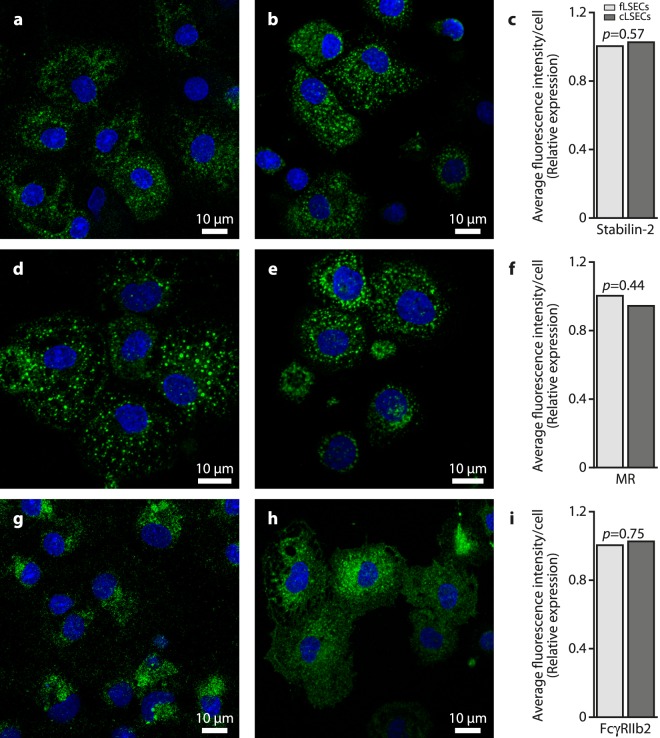
Expression of main endocytosis receptors by fLSEC and cLSECs. In all micrographs, the fLSECs are shown in the left panel and cLSECs in the right panel. The cultures were fixed with paraformaldehyde, permeabilized with Triton X, and immune labeled with antibodies against stabilin-2 (**a** and **b**), mannose receptor (MR) (**d** and **e**), and FcγRIIb2 (**g** and **h**). Positive immunolabeling was visualized with Alexa Fluor-488 secondary antibodies (green fluorescence). Cell nuclei were stained with DAPI (blue fluorescence). (**g**—**i**) The average fluorescence intensity per cell for each receptor protein was measured and the results expressed as relative expression, where the expression of the different markers in fLSECs equals 1. The *p* value is shown, which was calculated using the Excel two-tailed paired *t*-test assuming unequal variation. Statistical details are presented in Table 2 under Materials and Methods.

Just as fenestrations are the gold standard for intact ultrastructural LSEC-specific identity, the functional hallmark of these cells is their effective uptake of soluble macromolecules that are cleared via clathrin-mediated endocytosis^47^. Studies over the last couple of decades have established that the LSEC endocytic function relies mostly on the stabilin-1 and stabilin-2^48–50^, mannose receptor (MR)^10,51,52^, and Fc-gamma receptor IIb2 (FcγRIIb2)^53,54^. The expression of these receptors was tested in cultures of LSECs by immunofluorescence (Fig. 3). Total fluorescence intensity per cell was measured for each receptor protein staining, and we found no significant difference in the expression between the two groups (Fig. 3 right panel).

**Figure 4:**
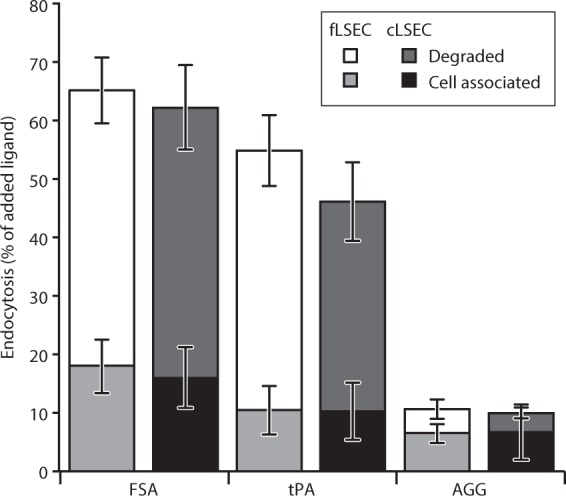
Endocytic ability of fLSEC and cLSECs. Confluent cultures of fLSEC and cLSECs were established in 24-well plates and incubated for 2 h at 37 °C with trace amounts of radiolabeled ligands for the main endocytosis receptors (^125^I-FSA for stabilin1/2, ^125^I-tPA for MR, and ^125^I-AGG for FcγRIIb2). At the end of the incubation time, the amount of cell association radioactivity and degraded radioactivity was measured in the cells and spend medium. Bars represents mean ± SD. Statistical details are presented in Table 2 under Materials and Methods.

In fully functional LSECs, the macromolecules that are recognized by these receptors are rapidly trafficked to, and efficiently degraded in the endo-lysosomal compartments. Here, we have tested the endocytic ability of cLSECs and compared it with the endocytic ability of fLSECs, by challenging the cultures with radiolabeled formaldehyde-treated serum albumin (^125^I-FSA), tissue plasminogen activator (^125^I-tPA), and aggregated gamma globulin (^125^I-AGG), ligands specifically recognized by stabilin1/2, MR, and FcγRIIb2, respectively^11,54–56^ (Fig. 4). This assay allows precise quantification of the amount of ligand that is taken up by the cells. We found that cLSECs had virtually identical uptake and degradation ability as fLSECs based on all three endocytosis receptors tested.

## Conclusion

Here, we have established and optimized a method for cryopreservation of rat LSECs. This cryopreservation method is very simple and reproducible, inexpensive, and readily available at any time point in any laboratory, without having to spend time and resources for expensive cryopreservants and method optimization. Cryopreserving freshly isolated rat LSECs using this method results in unchanged phenotype upon thawing and culturing. The ability to cryopreserve fully functional LSECs will facilitate a significant increase in research using these cells, reducing the number of animals and costs associated with cell isolation, and enable experiments to be conducted within the time frame of a regular working day. Studying LSECs and their implications in most liver diseases^18^ is important for our understanding of the natural progress of these diseases, and has the prospect of making LSECs an attractive therapeutic target. Moreover, cLSECs provide an ideal cell type for toxicology studies and designing pharmacological strategies, since by utilizing fully functional cLSECs one can avoid the limitations with batch-to-batch variability in response to drug therapy, especially when the cells originate from animals with rare conditions or liver diseases.

## Materials and Methods

### Materials and Reagents

Collagenase P was from Worthington Biochemical (Lakewood, NJ). Human serum albumin (HSA) was from Octapharma (Ziegelbrucke, Switzerland). Culture medium RPMI 1640, supplemented with 20 mM sodium bicarbonate, 0.006% (wt/vol) penicillin, and 0.01% (wt/vol) streptomycin, phosphate buffer saline (PBS), bovine serum albumin (BSA), and fetal bovine serum (FBS) were from Sigma-Aldrich, Oslo, Norway. Human fibronectin was purified from human plasma by affinity chromatography on Gelatin Sepharose 4B as described by the manufacturer. Sephadex G-25 (PD-10 columns) and Percoll were from Amersham Biotech (Uppsala, Sweden). Carrier-free Na^125^I was from Perkin-Elmer Norge (Oslo, Norway), and 1,3,4,6-tetrachloro-3α, 6α-diphenylglycoluril (Iodogen) was from Pierce Chemical (Rockford, IL). Formaldehyde-treated bovine serum albumin (FSA) was prepared as described^57^. Aggregated gamma globulin (AGG) was prepared from human normal immunoglobulin (100 mg/ml); Baxter, Vienna, Austria) by diluting it 1:9 with PBS and incubation at 63 °C in a water bath for 1 hour. Tissue plasminogen activator (tPA) was from American Diagnostica Inc., Stamford, CT, USA. Polyclonal rabbit anti rat stabilin-2 antibody was prepared as described^50^. Polyclonal goat anti human mannose receptor (MR) and polyclonal goat anti human FcγRIIb were from R&D Systems (Minneapolis, MN, USA). Both antibodies against human MR and FcγRIIb also react with rat specimens. Rabbit nonimmune IgG and goat serum were from Sigma-Aldrich, Oslo, Norway. DRAQ5 was from Biostatus Limited (Leicestershire, UK). Vybrant DiO, CellMask Deep Red, Alexa Fluor-488 goat anti rabbit IgG, and Alexa Fluor-488 rabbit anti goat IgG secondary antibodies were from ThermoFisher Scientific, Oslo, Norway.

### Labeling procedures

FSA, tPA and AGG (50 mg in 0.1 ml PBS) were labeled with carrier-free Na^125^I in a direct reaction employing Iodogen as oxidizing agent, as described^58^. Radiolabeled ligands and free iodine were separated by gel filtration on PD-10 columns equilibrated with PBS containing 1% human serum albumin. The specific activities were 3.3–5.1 × 10^6^ cpm/µg for FSA, 1.3–1.6 × 10^6^ cpm/µg for tPA, and 2.8–3.2 × 10^6^ cpm/µg for AGG.

### Isolation and culture of rat liver sinusoidal endothelial cells

Sprague Dawley, Crl:CD(SD), male rats (Charles River, Sulzfeld, Germany) were group housed (2–3 rats per cage) in conventional Eurostandard type IV cages with aspen bedding (Tapvei, Estonia) and with nesting material (Sizzelnest, Datesand, UK), rat tunnels (Scanbur, Norway) and aspen chew blocks (Scanbur, Norway) as environmental enrichment. The rats were housed under controlled environmental conditions (21 °C ± 1°, relative humidity 55% ± 5% and 12-hour light/12-hour dark cycle). They were fed a standard chow *ad libitum* (RM1-E, Special Diet Service, UK) and tap water ad libitum. The rats (body weight 250–350 g) were anesthetized with a mixture (ZRF-mix) of zolazepam/tiletamine hydrochloride 12.9/12.9 mg/ml (Zoletil forte vet, Virbac, Norway), xylazine 1.8 mg/ml (Rompun, Bayer Nordic, Norway) and fentanyl 10.3 µg/ml (Actavis, Norway). The experimental protocols were approved by the Norwegian Food Safety Authority (approval ID: 8455). Animal handling performed at the University of Bielefeld were approved by and carried out according to local authorities (Bezirksregierung Düsseldorf) and international guidelines. Rat LSECs were isolated and purified from anesthetized rats by Percoll separation and selective adherence^35^. Briefly, the liver was perfused with collagenase, and the resulting single cell suspension was subjected to velocity and density centrifugations in Percoll gradients to produce purified suspensions of hepatocytes and nonparenchymal cells (NPCs). The NPC suspension was a mixture of Kupffer cells (KCs) and LSECs, and essentially devoid of hepatocytes, red blood cells, and debris. The NPC suspension was seeded directly on plastic in three 25 cm^2^ culture dishes (Nunc, Roskilde, Denmark). Following a 45 min incubation at 37 °C, only KCs attached and spread onto the substrate, resulting in a highly enriched LSEC fraction in the supernatant. LSEC preparations were between 95% and 98% pure. The usual contaminants have been previously reported to be Kupffer cells (KCs; CD163-positive), and Stellate cells (SCs; identified by their content of autofluorescent vitamin A)^11^. In this study, non-fenestrated cells identified by SEM were considered contaminants. Immediately after isolation, a fraction of the cells was seeded in the respective experimental conditions, and the remaining cells dispensed in cryotubes for cryopreservation.

### Freezing, thawing and culturing rat LSECs

Following the last step of isolation, the cells in suspension were counted using a hemocytometer and the viability assessed by Trypan Blue exclusion (>95%). The cells were then split into two fractions and pelleted by centrifugation for 8 min at 300 G, 4 °C. The supernatant was decanted without disturbing the cell pellet. Each cell pellet was resuspended at a final concentration of 4 × 10^6^ LSECs/ml in the freezing media as described in Table 1. The solution was then dispensed into Nunc CryoTubes (Sigma-Aldrich, Oslo, Norway), and the tubes immediately placed in Mr. Frosty cryo container (ThermoFisher Scientific, Nalgene, Oslo, Norway). The container was transferred to −80 °C until the next day when the cryotubes were transferred to liquid nitrogen for long term storage.

**Table 1:** Freezing media for cryopreservation of freshly isolated rat LSECs.

Freezing medium containing 70% RPMI, 20% FBS, and 10% DMSO	Freezing medium containing 5% RPMI, 90% FBS, and 5% DMSO
Resuspend the cells in 1–2 ml cold RPMI	Resuspend the cells in 1–2 ml cold FBS
Add cold RPMI to the final calculated volume	Add cold RPMI
Add cold FBS	Add cold FBS to the final calculated volume
Resuspend the cells using a 5 ml pipette	Resuspend the solution using a 5 ml pipette
Dropwise, add the DMSO while rotating the tube containing the cells	Dropwise, add the DMSO while rotating the tube containing the cells
Resuspend the solution using a 5 ml pipette	Resuspend the solution using a 5 ml pipette
Dispense the final solution in 1 ml per cryotube	Disperse the final solution in 1 ml per cryotube

For thawing the cLSECs, the cryotubes were retrieved from the liquid nitrogen tank and immediately placed into a 37 °C water bath. The tubes were swirled until only a small bit of ice was visible. Immediately, the cell suspension was dropwise added to a centrifuge tube containing 40 ml pre-warmed serum-free RPMI. After centrifugation for 8 min at 300 G, the supernatant was discarded and the pelleted cells gently resuspended in serum-free RPMI. Cell number and viability was assessed prior seeding the cells in the respective experimental conditions.

### Scanning electron microscopy (SEM)

Cultures of fLSECs and cLSECs from the same isolation were established in serum-free RPMI-1640 at a density of 0.1 × 10^6^ cells/cm^2^ on fibronectin coated plastic 6-well culture plates. The concentration of the fibronectin used was 0.2 mg/ml, and the coating was done using just enough volume to completely cover the surface area. After 10 min of incubation at RT, the fibronectin was washed off with PBS and cells seeded. The cells were fixed overnight in McDowell’s or 4% formaldehyde (FA), 2.5% glutaraldehyde (GA) in cacodylic buffer. After washes with PBS, the bottom of the dishes containing the cells were cut off and treated with 1% tannic acid in 0.15 mol/l cacodylic buffer, 1% OsO_4_ in 0.1 mol/l cacodylic buffer, dehydrated in ethanol, and incubated in hexamethyldisilazane (Sigma-Aldrich, Oslo, Norway), before coating with 10-nm gold/palladium alloys. Large field of view containing several cells, and high resolution SEM images of individual cells were acquired to assess cell size, fenestrations size and porosity. The iTEM software (Olympus, Asker, Norway) was used for measuring the cell diameter, while measurements of fenestration size and porosity were done using the public domain software Fiji (https://fiji.sc)^59^.

### Fluorescence microscopy

Cultures of fLSECs and cLSECs from the same isolation were established in serum-free RPMI-1640 at a density of 0.1 × 10^6^ cells/cm^2^ on fibronectin coated 13 mm diameter #1.5 glass coverslips (VWR, Oslo, Norway) and #1.5 glass bottom dishes (MatTek, Ashland, MA, USA). Following attachment and spreading of the cytoplasm, the cells were either fixed and immunolabeled for confocal microscopy, or stained and observed live by structured illumination microscopy (SIM). For immunolabeling, the cells were washed and fixed in 4% FA for 15 min at room temperature. After a blocking step of 30 min with PBS containing 1% BSA, the cells were permeabilized in 0.03% Triton X-100 for 4 min and immune labeled by antibodies against stabilin-2, MR, or FcγRIIb as described^50,60^. Rabbit nonimmune IgG and goat serum were used as negative controls. The Positive staining was visualized by using secondary antibodies tagged with Alexa Fluor-488 and DRAQ5 for nuclear staining. Specimens were examined using a Zeiss Laser Scanning Microscope 780 Meta (Carl Zeiss Microimaging GmbH, Göttingen, Germany) with a water-immersion Apochromat 40x/1.4 objective lens. For live super resolution imaging of the fenestrations, the cells were either stained with Vybrant DiO (1:200 in serum-free RPMI) or with CellMask Deep Red (1:5000 in serum-free RPMI) for 10 minutes and immediately imaged using a commercial super-resolving structured illumination microscope (DeltaVision/OMXv4.0 BLAZE, GE Healthcare) equipped with a 60X 1.42NA oil-immersion objective (Olympus). 3D-SIM images stacks of 1 μm were acquired with a *z*-distance of 125 nm and with 15 raw images per plane (five phases, three angles). Raw datasets were computationally reconstructed using SoftWoRx software (GE Healthcare). For clarity of display, linear changes were made to brightness and contrast of the images. Total fluorescence intensity per cell was measured using the Fiji software.

### Endocytosis and degradation assay

For quantitative studies of endocytosis and degradation, fully confluent cultures of fLSECs and cLSECs (approx. 0.2–0.25 × 10^6^ cells/cm^2^) established in 24-well culture dishes coated with fibronectin were incubated in 0.2 ml serum-free RPMI containing 0.1% human serum albumin and 2–3 × 10^4^ cpm ^125^I-FSA,^125^I-tPA, or ^125^I-AGG. After 2 h of incubation at 37 °C, the amount of degraded ligands was measured by collecting the spent medium together with one wash volume of 0.5 mL PBS. TCA (0.75 ml, 20%) was added to precipitate intact phages. The amount of TCA-soluble radioactivity measured in the supernatant after centrifugation represented degraded ligands. To determine the amount of cell bound and internalized ligands, the cells were lysed in 0.1% sodium dodecyl sulfate (SDS). The radioactivity was measured using a Cobra II, Auto-Gamma detector (Packard Instruments, Laborel, Oslo, Norway). The amount of non-specific binding and free ^125^I in cell-free wells was subtracted. The total endocytosis represents the sum of cell-associated and acid-soluble radioactivity.

### Statistics

Table 2 summarizes the number of animals, the data, and the statistics done for the experiments included in this study. Measurements of cell size, fenestration size, and porosity were done using the SEM images. Only cells with clearly identifiable cellular borders were used for measurement of cell diameters. Fenestration size and porosity were assessed in SEM images from each cell culture selected from different areas (up, right, down, left and middle areas). Fenestrations were defined as open pores with diameters between 50–300 nm. Porosity was defined as the sum area of fenestrations per total area of the cell in the micrograph. Gaps were defined as holes with a diameter larger than 300 nm. Fiji software was used to identify and measure the area of all fenestrations (circularity 0.6–1) and gaps (circularity 0.1–1). Total fluorescence intensity per cell for each receptor staining was measured using Fiji after adjusting for background fluorescence. The results are expressed as relative expression, where the expression of the different markers in fLSECs equals 1. Comparison between the two groups was performed using the Excel two-tailed paired *t*-test assuming unequal variation. Differences were considered significant if *p* < 0.05.

**Table 2:** Summary of the repeats for each experiment. Each experiment was performed independently, and in parallel, using cells from 2–3 separate rats/cryotubes.

Result	No. of rats(separate cryotube)	Repeats	Statistical variation	Figure
Cell size			SD	1c,d
fLSECs	5	27 images/449 cells		
cLSECs	3	13 images/281cells		
fLSECFenestration diameter/size distribution/porosity/gaps	4	44 images/137 cells78120 fenestrations6208 gaps	SEM	2a–d
cLSECFenestration diameter/sizedistribution/porosity/gaps	5	28 images/44 cells26731 fenestrations2107 gaps	SEM	2a–d
Immunocytochemistry Stabilin 2	3		Two tailed paired *t-*test assuming unequal variation	3
fLSECs		103 cells		
cLSECs		79 cells		
Mannose receptor				
fLSECs		103 cells		
cLSECs		97 cells		
FcγRIIb				
fLSECs		97 cells		
cLSECs		66 cells		
Endocytosis assay				
^125^I-FSA		2–3 repeats (2–3 wells with cells +1 well-cell free control) for each time an experiment was performed		4
fLSECs	7			
cLSECs	5		SD	
^125^I-tPA				
fLSECs	3		SD	
cLSECs	5			
^125^I-AGG				
fLSECs	4			
cLSECs	4		SD	
